# Adolescent Metabolic Syndrome Risk Is Increased with Higher Infancy Weight Gain and Decreased with Longer Breast Feeding

**DOI:** 10.1155/2012/478610

**Published:** 2012-07-05

**Authors:** Kim Khuc, Estela Blanco, Raquel Burrows, Marcela Reyes, Marcela Castillo, Betsy Lozoff, Sheila Gahagan

**Affiliations:** ^1^Division of Child Development and Community Health, University of California, San Diego, 9500 Gilman Drive, La Jolla, CA 92093-0927, USA; ^2^Institute of Nutrition and Food Technology (INTA), University of Chile, El Líbano 5524, 138-11 Santiago, Chile; ^3^Center for Human Growth and Development and Department of Pediatrics and Communicable Diseases, University of Michigan, 300 North Ingalls, 10th Floor, Ann Arbor, MI 48109-5406, USA

## Abstract

*Background*. Prevalence of the metabolic syndrome is increasing in pediatric age groups worldwide. Meeting the criteria for the metabolic syndrome puts children at risk for later cardiovascular and metabolic disease. *Methods*. Using linear regression, we examined the association between infant weight gain from birth to 3 months and risk for the metabolic syndrome among 16- to 17-year-old Chilean adolescents (*n* = 357), accounting for the extent of breastfeeding in infancy and known covariates including gender, birth weight, and socioeconomic status. *Results*. Participants were approximately half male (51%), born at 40 weeks of gestation weighing 3.5 kg, and 48% were exclusively breastfed for ≥90 days. Factors independently associated with increased risk of metabolic syndrome in adolescence were faster weight gain in the first 3 months of life (*B* = 0.16, *P* < 0.05) and male gender (*B* = 0.24, *P* < 0.05). Breastfeeding as the sole source of milk for ≥90 days was associated with significantly decreased risk of metabolic syndrome (*B* = −0.16). *Conclusion*. This study adds to current knowledge about early infant growth and breastfeeding and their long-term health effects.

## 1. Introduction

As the prevalence of obesity in children and adolescents has escalated worldwide, signs of the metabolic syndrome (MetS) are increasingly observed in the pediatric age range [1]. MetS refers to a cluster of abnormal physical examination and laboratory findings, including high waist circumference, serum triglyceride, serum glucose, and blood pressure, and low-serum HDL-cholesterol. These findings synergistically relate to risk for developing type 2 diabetes and cardiovascular diseases (CVDs), including coronary artery disease and stroke [2–4]. In a study of a representative sample of US 12- to 19-year-old, 8.6% met criteria for MetS; Hispanic youth had a higher prevalence (11.2%) than white (8.9%) or black adolescents (4.0%) [2]. Children who meet the criteria are at increased risk for CVD in adulthood [5]. Focusing on the MetS during the pediatric period is expected to lead to early-prevention strategies for diabetes and CVD [5]. 

A large body of evidence suggests that metabolic programming can occur early in life [6–8]. Early-life risk factors include low birth weight and rapid postnatal weight gain. Breastfeeding including duration and dose appears to offer protection for obesity, type 2 diabetes, the MetS, and CVD [9–12]. In fact, the time immediately before and after birth may be a sensitive period related to metabolic and cardiovascular risk [13]. Rapid post-natal weight gain is associated with increased risk for obesity, type 2 diabetes and hypertension in young adulthood [13–18]. Infant weight gain, especially in the first 3 months, may be more important than birth weight as a predictor of later health outcomes [13]. Adolescent MetS has previously been found to be associated with infancy growth in the setting of a developed country [19]. 

Much of the work on fetal origins of disease has been done in developed countries beginning with Barker's work in England [7]. Research in low- to middle-income countries is needed to further delineate the roles of biology and environment related to early-life risk for cardiovascular disease and related conditions. Our cohort of low- to middle-income Chilean adolescents, studied since infancy, provides a special opportunity to address these research questions, especially because the participants were born during a period of rapid nutritional and economic transition in Chile. This transition was characterized by economic progress that led to increased consumption of calories, fat, animal protein, and processed foods, and increased mortality from noncommunicable chronic diseases [20]. The aims of this study were to examine the association between infant weight gain from birth to 3 months and risk for the MetS in mid-adolescence, accounting for the extent of exclusive breastfeeding in infancy and covariates known to be associated with infant growth and the MetS, gender, birth weight, and socioeconomic status (SES).

## 2. Methods

### 2.1. Cohort

This is an observational cohort study involving adolescents who were enrolled as infants in a randomized controlled trial of iron supplementation to prevent iron deficiency anemia. Infants were enrolled from 1991–1996 in Santiago, Chile; 1657 infants completed the preventive trial at 1 year. The inclusion criteria for the preventive trial were infant birth weight of 3 kg or more, with no birth complications, major congenital abnormalities, or prior iron therapy. Due to a highly successful national breastfeeding campaign, all but 8 infants in the cohort were initially breastfed. Infants were randomly assigned to low or high iron supplementation, or usual nutrition (no added iron). A more detailed description of randomization techniques, sampling, and entrance and exclusion criteria is published elsewhere [21]. The participants have been involved in follow-up studies at 5, 10, and 16 years. At 16 years, the participants from the longitudinal cohort were invited to enroll in a study of adolescent obesity and cardiovascular risk. We report on the first 384 studied from a randomly selected sample of the original cohort evaluated at 16 years between May 2009 and January 2011. Complete data from the infancy and adolescent waves were available for 357 of the 384 adolescents. Infancy variables (birth weight, weight at 3 months, and gestational age) did not differ between the 357 studied and the original 1657 infant participants. Our analytic sample was more likely to receive bottle supplementation before 90 days, compared to the larger cohort (52% versus 45%, *P* < 0.05). The longitudinal study has been approved by the institutional review boards of the University of Michigan, Ann Arbor USA; Institute of Nutrition and Food Technology, University of Chile, Santiago, Chile for each wave of study; by the University of California, San Diego, for the 16-year study of obesity and cardiovascular risk.

### 2.2. Infancy Data

We included the following infancy measures: gender, weight measured at birth and at 3 months, and date of the first supplemental bottle. Maternal education was used as a proxy for SES. Mother's prepregnancy BMI was calculated from measured height and self-report of prepregnancy weight. Data on pre-pregnancy weight was not collected for the infancy study. During the 10-year wave of data collection, mothers reported their pre-pregnancy weight; it was highly correlated with their actual weight 10 years later. 

### 2.3. Adolescent Data

Adolescents were assessed between 16 and 17 years during the fourth wave of the longitudinal research study (infancy, 5 years, 10 years, and 16-17 years). Height (cm), weight (kg), waist and hip circumference (cm), and blood pressure (mm Hg) were measured by a physician-investigator at the nutrition research center. Standardized procedures [22] were used to measure weight to the closest 0.1 kg, using a SECA scale, and height to the closest 0.1 cm, using a Holtain stadiometer. Measurements were taken twice, with a third measurement if the difference between the first two exceeded 0.3 kg for weight and 0.5 cm for height. Fasting serum triglyceride, cholesterol, and glucose levels were performed. Serum glucose concentration (mg/dL), triglycerides (mg/dL), and cholesterol (mg/dL) levels were determined using an enzymatic-colorimetric test (QCA S.A., Amposta, Spain). Using a standardized questionnaire, parents reported family history of type 2 diabetes, hypertension, dyslipidemia, and heart attack before the age of 60, in first-degree relatives. 

### 2.4. Statistical Analysis

Infant weight gain in the first 3 months was calculated as weight gain velocity over the first 3 months (91.3 days): (weight [kg] at 3-month birth weight [kg])/(age at 3-month measurement ∗ 91.3 days). Extent of breastfeeding was assessed as a dichotomous variable representing breastfeeding without bottle supplementation for less than 90 days, compared to 90 days or more. Data was not available on introduction of complementary foods. Maternal education was assessed as a continuous measure (median for sample = 10 years). We constructed a metabolic syndrome risk *z*-score according to the work of Brage and colleagues [23]. The following variables were converted to *z*-scores: the reciprocal of the HDL value, the mean of the systolic and diastolic blood pressure measurements, waist circumference, fasting serum triglyceride, and glucose. We obtained a continuous, normally distributed metabolic risk *z*-score by averaging these 5 values. 

For descriptive statistics, continuous variables were expressed as median and interquartile ranges and categorical variables as frequencies. We evaluated cardio/metabolic risk factors and overall prevalence of the MetS according to International Diabetes Federation definition [24]: waist circumference ≥94 cm for boys and ≥80 cm for girls, plus any two of the following four factors: triglycerides ≥1.7 mmol/L, HDL-cholesterol <40 mg/dL in males and <50 mg/dL in females, systolic BP ≥130 or diastolic BP ≥85 mm Hg, fasting plasma glucose ≥100 mg/dL. BMI percentile was described according to CDC standards. 

We used SPSS for Windows version 18.0 (Chicago, IL, USA), a *P* value of <0.05 denoted statistical significance. Multiple linear regression models were used to determine the relationship between change in weight (kg) in the first 3 months and metabolic syndrome risk *z*-score, adjusting for extent of breastfeeding and the following covariates: birth weight, gender, SES, age, mother's age at birth of infant, mother's pre-pregnancy BMI, and family history of type 2 diabetes, dyslipidemia, and heart attack. We tested the full model and then, using backward elimination, removed each variable that was not significantly related to the outcome in the model based on a significant *P* value of <0.05. As the sample came from an iron-deficient anemia prevention trial, we tested whether iron-deficient anemia during the first year of life or iron supplementation were significant covariates in our models. Neither variable showed significant relationship in the models and were thus removed from the final models. 

## 3. Results

Participants were assessed at a mean age of 16.6 years. Males and females represented about 51% and 49% of the sample, respectively. Participants had been born at 40 weeks of gestation weighing 3.5 kg, on average, and 48% were exclusively breastfed for ≥90 days. The median BMI percentile was 68.7 with 15.2% in the obese range and 10.4% met criteria for MetS.  Table 1 describes infant and family background characteristics by gender of the 357 participants in infancy and adolescence. Cardio/metabolic risk factors are also described. Males had higher birth length, weight at three months, and higher weight gain between birth and 3 months compared to females. Males also had significantly lower HDL cholesterol and higher blood pressure values (systolic and diastolic), glucose and MetS risk *z*-scores than females. There were no significant differences between males and females in gestational age, birth weight, maternal education, exclusive breastfeeding for ≥90 days, and prevalence of the MetS. 

The multiple variable linear regression models (full and final) are shown in Table 2. The final model revealed that weight gain over the first three months was associated with an increased MetS risk score at 16-17 years, taking into account extent of breastfeeding and gender (*B* = 0.16, 95%  CI = 0.04, 0.27, *P* < 0.05). Introduction of the first bottle at 90 days or after was related to a lower MetS risk score in adolescence (*B* = −0.16, 95%  CI = −0.29, −0.04, *P* < 0.05), taking into account other covariates. Additionally, being male was associated with an increased MetS risk score in the model (*B* = 0.24, 95%  CI = 0.11, 0.37, *P* < 0.05). The final model explained 9% of the variance in MetS risk.

## 4. Discussion

We examined weight gain in the first 3 months of life and timing of bottle supplementation related to MetS risk at 16 years. In both sexes, adolescents who had more rapid weight gain during the first 3 months of infancy had higher adolescent MetS risk scores compared to those who gained less weight in early infancy. The association of weight gain with MetS risk is consistent with findings from a study addressing the same question, in a Scandinavian country [19]. Infancy weight gain has previously been associated with later obesity in childhood and adulthood [15, 18, 25, 26]. In addition, especially for low-birth-weight infants, more rapid early weight gain, sometimes called catch-up growth, has been related to higher risk of developing type 2 diabetes and/or cardiovascular disease [10]. Contrary to our findings, a Finnish study found that infants who had low weight gain in the first 6 months had higher risk for development of glucose intolerance, an effect that was greater for those with low birth weight [27]. Since our cohort excluded infants with birth weights below 3 kg, it is clear that the association we find between infant weight gain and adolescent MetS risk is independent of low birth weight. Furthermore, this association did not depend on family history of conditions related to the MetS such as type 2 diabetes, dyslipidemia, or myocardial infarction.

There is accumulating evidence that breastfeeding offers some protection related to the development of obesity, and that the effect may be “dose-dependent” [28, 29]. Because breastfed infants gain weight more slowly over the first year compared to formula-fed infants [30, 31], infant weight gain may pertain to the mechanism that decreases obesity risk in those who were breastfed. Having been breastfed has also been associated with lower risk for hypercholesterolemia, hypertension, diabetes, glucose intolerance, and insulin resistance [9–12]. To our knowledge, no other study has shown an association between breastfeeding and MetS risk in adolescence. Importantly, the significant effects of weight gain and breastfeeding were independent, suggesting that the effect of breastfeeding on MetS risk was not mediated by early infancy weight gain.

We do not know why males had higher MetS risk scores compared to females, but they had marginally significant higher birth weights and gained more weight in the first 3 months. Nonetheless, the effect of gender on MetS was independent of birth weight and infancy weight gain. This finding is consistent with higher prevalence rates of MetS in men compared to women in Chile [32]. In US adolescents, males are also more likely to have clustering of metabolic syndrome risk factors compared to girls [2]. However, in the Scandinavian study of infant weight gain and the MetS, male gender was not related to higher MetS risk [19], even though boys were similarly heavier at birth and gained more in infancy than girls. This suggests that the effect of gender is related to context rather than biology.

Our study has several limitations. The cohort was enrolled from a low- to middle-income community in Santiago, Chile, during a period of economic and nutritional transition. The setting and the fact that children with birth weights under 3 kg were not included limits generalizability. The study also has many strengths. The context of economic growth, high rates of breastfeeding, and nutritional support for infants allowed us to assess a sample where malnutrition was not a confounding factor. The longitudinal study took place at a nutrition research center allowing for detailed anthropometric measurement during infancy and the adolescent wave of data collection. Other strengths of the study include prospective data collection including monthly anthropometry in infancy and breastfeeding data collected from 4 to 12 months. In addition, the adolescent data collection included family history of diabetes, hypertension, elevated cholesterol, and heart attack.

## 5. Conclusion

In conclusion, this study adds to the current knowledge about early infant growth and breastfeeding and their long-term health effects. Higher infant weight gain was associated with increased MetS risk, whereas longer duration of exclusive breastfeeding was protective in healthy adolescents living in a rapidly developing country. Considering the increasing prevalence of the MetS in younger age groups and associations between the MetS and later disease, the replication and validation of these findings in different contexts is warranted. 

## Figures and Tables

**Table 1:** Background characteristics of study participants by gender.^†^

	Males (*n* = 181)	Females (*n* = 176)	*P* value^∗^
Infancy characteristics			
Gestational age (weeks)	40 (38–40)	40 (39-40)	0.22
Birth weight (kg)	3.50 (3.28–3.78)	3.44 (3.22–3.72)	0.05
Birth length (cm)	51.0 (50.0–52.0)	50.0 (49.0–51.5)	<0.01
Weight at 3 months (kg)	6.43 (6.00–6.99)	6.00 (5.61–6.40)	<0.01
Weight gain 0–3 months (kg)	2.85 (2.43–3.28)	2.42 (2.15–2.75)	<0.01
Total years of maternal education	10 (8–12)	10 (8–12)	0.52
Exclusively breastfed ≥90 days	45.9	50.6	0.37
Adolescent characteristics and metabolic risk factors^‡^			
Age (years)	16.6 (16.6–16.9)	16.7 (16.6–16.9)	0.15
BMI percentile	64.0 (38.6–88.2)	74.9 (46.0–89.9)	0.10
Waist circumference (cm)	78.2 (73.3–87.9)	79.7 (72.2–88.1)	0.83
Elevated percentage	16.0	49.0	<0.01
Triglycerides (mg/dL)	71.6 (55.8–99.2)	73.0 (57.2–104.4)	0.77
Elevated percentage	7.2	8.0	0.78
HDL cholesterol (mg/dL)	34.1 (28.5–40.5)	40.0 (32.5–48.3)	<0.01
Low HDL (%)	28.2	24.0	0.29
Systolic blood pressure (mm Hg)	115 (107–125)	107 (102–114)	<0.01
Elevated percentage	8.3	4.4	0.09
Diastolic blood pressure (mm Hg)	70 (67–76)	68 (61–71)	<0.01
Elevated percentage	3.9	0.6	0.03
Glucose (mg/dL)	90 (84–96)	86 (80–92)	<0.01
Elevated percentage	11.6	9.1	0.44
MetS risk *z*-score	0.01 (−0.23–0.40)	−0.26 (−0.53–0.02)	<0.01
MetS prevalence	11.6	9.1	0.45
Family factors			
Family history of type 2 diabetes	15.3	14.4	0.82
Family history of hypertension	42.3	40.6	0.76
Family history of dyslipidemia	33.6	45.5	0.04
Family history of heart attack	8.3	5.1	0.29

^†^Values are in median (interquartile range) or percentage. ^∗^Statistical tests are either Mann-Whitney *U* or Chi-square.

^‡^According to the International Diabetes Federation definition [24].

**Table 2:** Linear regression models to determine adjusted associations with adolescent MetS risk (*n* = 357).

	Initial model		Final model	
	*β*	95% CI	*β*	95% CI
Change in weight 0–3 months (kg)	0.15	−0.01, 0.31	0.16	0.04, 0.27
Exclusively breastfed ≥90 days	−0.19	−0.36, −0.03	−0.16	−0.29, −0.04
Male	0.23	0.05, 0.01	0.24	0.11, 0.37
Birth weight (kg)	0.09	−0.14, 0.04		
Maternal education	0.01	−0.01, 0.01		
Mother's age	0.00	−0.01, 0.01		
Maternal pre-pregnancy BMI	0.02	−0.01, 0.05		
Family history of type 2 diabetes	0.10	−0.10, 0.27		
Family history of dyslipidemia	0.00	−0.17, 0.18		
Family history of heart attack	−0.06	−0.23, 0.10		
Ever iron deficiency in infancy	−0.09	−0.35, 0.16		
Iron supplemented in infancy	0.02	−0.16, 0.20		
